# Polarized Entry of Human Parechoviruses in the Airway Epithelium

**DOI:** 10.3389/fcimb.2018.00294

**Published:** 2018-08-22

**Authors:** Eveliina Karelehto, Cosimo Cristella, Xiao Yu, Adithya Sridhar, Rens Hulsdouw, Karen de Haan, Hetty van Eijk, Sylvie Koekkoek, Dasja Pajkrt, Menno D. de Jong, Katja C. Wolthers

**Affiliations:** ^1^Laboratory of Clinical Virology, Department of Medical Microbiology, Academic Medical Center, University of Amsterdam, Amsterdam, Netherlands; ^2^Department of Pediatric Infectious Diseases, Academic Medical Center, Emma's Children's Hospital, Amsterdam, Netherlands

**Keywords:** human parechovirus, airway epithelium, basal cell, host response, basolateral infection

## Abstract

Human parechoviruses (HPeVs), a poorly studied genus within the *Picornaviridae* family, are classified into 19 genotypes of which HPeV1 and HPeV3 are the most often detected. HPeV1 VP1 C terminus contains an arginine-glycine-aspartic acid (RGD) motif and has been shown to depend on the host cell surface αV integrins (αV ITGs) and heparan sulfate (HS) for entry. HPeV3 lacks this motif and the receptors remain unknown. HPeVs can be detected in patient nasopharyngeal and stool samples, and infection is presumed to occur after respiratory or gastro-intestinal transmission. HPeV pathogenesis is poorly understood as there are no animal models and previous studies have been conducted in immortalized monolayer cell cultures which do not adequately represent the characteristics of human tissues. To bridge this gap, we determined the polarity of infection, replication kinetics, and cell tropism of HPeV1 and HPeV3 in the well-differentiated human airway epithelial (HAE) model. We found the HAE cultures to be permissive for HPeVs. Both HPeV genotypes infected the HAE preferentially from the basolateral surface while the progeny virus was shed toward the apical side. Confocal microscopy revealed the target cell type to be the p63^+^ basal cells for both viruses, αV ITG and HS blocking had no effect on the replication of either virus, and transcriptional profiling suggested that HPeV3 infection induced stronger immune activation than HPeV1. Genotype-specific host responses may contribute to the differences in pathogenesis and clinical outcomes associated with HPeV1 and HPeV3.

## Introduction

Parechovirus A species within the *Picornaviridae* family comprises of 19 genotypes, of which human parechovirus 1 and 3 (HPeV1 and HPeV3, respectively) are the most often detected in clinical samples (Harvala et al., 2010; Romero and Selvarangan, 2011). HPeVs are major human pathogens, typically causing respiratory, gastrointestinal, and febrile illness in young children (Harvala et al., 2010; Romero and Selvarangan, 2011). When compared to HPeV1, HPeV3 infects younger patients and often causes more severe diseases, such as sepsis-like illness or central nervous system (CNS) infections. The reason for these differences remains elusive (Benschop et al., 2006; Romero and Selvarangan, 2011).

The HPeV single-stranded positive-sense RNA genome coding region, flanked by 5′ and 3′ untranslated regions, is translated into a single polyprotein and subsequently cleaved into three capsid proteins (VP0, VP3, and VP1) and seven non-structural proteins (2A-C and 3A-D). HPeV1 contains a well-known integrin-recognition sequence, the arginine-glycine-aspartic acid (RGD) motif, in the VP1 C terminus, while HPeV3 lacks this motif (Stanway et al., 1994; Ito et al., 2004). Previous studies have identified three αV integrin (αV ITG) heterodimers as HPeV1 receptors (αVβ1, αVβ3, and αVβ6) (Triantafilou et al., 2000; Joki-Korpela et al., 2001; Seitsonen et al., 2010; Merilahti et al., 2016b). Heparan sulfate (HS) has been proposed to act as an attachment receptor for HPeV1 (Merilahti et al., 2016a). To date, no HPeV3 receptors have been identified. Analogous to virus species in the closely related Enterovirus genus, HPeVs are detected in patient nasopharyngeal and stool specimens, and are assumed to initiate their replication in the host respiratory and intestinal tracts (Semler and Wimmer, 2002). However, it is not known whether the epithelium lining these anatomical sites can support HPeV replication nor which cell types are involved.

The air-liquid interface human airway epithelial (HAE) cell culture system is a well-characterized three-dimensional organotypic model that has been shown to closely mimic the structure and function of the *in vivo* parent tissue (Fulcher et al., 2005). In the current study, we used this model to investigate HPeV entry in one of the proposed primary replication sites, the airway epithelium. Given that integrins and heparan sulfate have been reported to be expressed at the basolateral surface of polarized epithelium (Erlinger, 1995; Esclatine et al., 2001; Lütschg et al., 2011), and that HPeV1 has been detected in respiratory patient samples more often than HPeV3 (Harvala et al., 2008), we hypothesized that HPeV replication efficiency would differ depending on the inoculation site and the HPeV genotype. We determined the polarity of infection and replication kinetics of HPeV1 and HPeV3 in the HAE model and performed immunofluorescence confocal microscopy analyses to characterize the HPeV cell tropism. Furthermore, we speculated that differences in the airway epithelium host response may contribute to the distinct clinical outcomes and performed transcriptome analyses to compare the HAE gene expression profiles induced by HPeV1 and HPeV3 infection.

## Materials and methods

### Viruses and cells

HPeV1 Harris strain was obtained from the National Institute of Public Health and the Environment (RIVM, Bilthoven). HPeV1 was cultured in HT29 cells (human colorectal adenocarcinoma; ATCC, Manassas, VA). HPeV3 152037 strain, isolated from a Dutch clinical specimen in 2001, was cultured in LLCMK2 cells (rhesus monkey kidney cell line, kindly provided by the Municipal Health Services, Rotterdam, the Netherlands). Enterovirus 71 (EV71) 91-480 and enterovirus 68 (EV68) 947 were kindly provided by the National Institute for Public Health and the Environment (Bilthoven, the Netherlands) and by Prof. van Kuppeveld, Utrecht University (Utrecht, the Netherlands), respectively. EV71 and EV68 were cultured in RD cells (human rhabdomyosarcoma; ATCC, Manassas, VA). All cell lines were maintained in Eagle's minimum essential medium (EMEM; Lonza, Basel, Switzerland) supplemented with 8% heat-inactivated fetal bovine serum (FBS; Sigma-Aldrich, St. Louis, MO), PEN-STREP (100 U/ml penicillin and 100 U/ml streptomycin; Lonza Bio Whittaker, non-essential amino acids (NEAA; ScienCell Research Laboratories, Carlsbad, CA) and L-glutamine (200 nM; Lonza, Basel, Switzerland). The 50% tissue culture infective dose (TCID50) of virus stocks was determined using the Reed and Muench method (Reed and Muench, 1938).

### Well-differentiated human airway epithelial (HAE) cell cultures (mucilair)

Nasal MucilAir HAE cell cultures from four individual donors (59701, 48401, 43601, 19702) were purchased from Epithelix Sàrl (Geneva, Switzerland) (Tapparel et al., 2013). Upon receiving the well-differentiated 24-well format transwell HAE inserts, they were cultured for 1 week at air-liquid interface before performing the infection experiments. The MucilAir culture medium (Epithelix Sàrl) was refreshed every 2–3 days.

### HAE infections

Donor 59701 HAE culture infections were performed twice using technical duplicates. To confirm results in additional donors, infection experiments were repeated once using donor 48401, 43601, and 19702 HAE cultures as biological replicates. Apical surfaces of the HAE cultures were washed once with Hank's Balanced Salt Solution (HBSS; ThermoFisher Scientific) and the culture medium refreshed prior to virus inoculation. HAE cultures were inoculated by adding 10^5^ TCID50 of HPeV1 or HPeV3 (or HBSS for mock) in a volume of 50 μl on the apical or basolateral surface. For basolateral infection, HAE inserts were inverted for the duration of inoculation. Virus inoculum was removed after 2-h incubation at 37°C, 5% CO_2_, and the inoculation surface of the HAE cultures was subsequently washed eight times with HBSS. Apical sampling was performed by adding 100 μl of HBSS on the HAE culture apical surface followed by a 10-min incubation at 37°C, 5% CO_2_, and collection. Basolateral samples were obtained by collecting 100 μl of the HAE culture medium. 100 μl of fresh culture medium was subsequently added to maintain the total culture medium volume at 600 μl per insert. EV68 was used as a control for apical infection and added only on the apical surface of the HAE cultures.

### HPeV detection by RT-qPCR and TCID50

RNA from 25 μl of the apical and basolateral samples was isolated by automatic extraction using the MagnaPure LC instrument (Roche Diagnostics, Almere, the Netherlands) and eluted in 50 μl. Forty micro liter of the RNA was reverse-transcribed and 5 μl of the cDNA was used for real-time quantitative PCR (RT-qPCR) targeting the 5' untranslated region of the HPeV genome (Benschop et al., 2008). In addition, apical and basolateral HAE samples were analyzed for the level of infectious HPeV by TCID50 titrations using the Reed and Muench method (Reed and Muench, 1938). HPeV1 and HPeV3 samples were titrated in the HT29 and LLCMK2 cell lines, respectively.

### Blocking of αV integrins and heparan sulfate

Apical surfaces of the HAE cultures were washed once with HBSS and the culture medium was refreshed prior to the blocking experiments. Host cell surface αV integrins were blocked by adding 50 μl of 15 μg/ml or 30 μg/ml of function-blocking human αV ITG mAb clone L230 (ALX-803-304-C100; Enzo Life Sciences, Inc. Farmingdale, NY) on the apical or basolateral surface of the HAE cultures. For basolateral blocking, HAE inserts were inverted for the duration of the pretreatment. mAb solution was removed after 1-h incubation at 37°C, 5% CO_2_, and replaced with mAb solution containing the virus inoculum. Virus binding to the host cell surface heparan sulfate was blocked by incubating the input virus with 5 mg/ml excess concentration of heparin sodium salt (H4784; Sigma Aldrich, St. Louis, MO) for 1-h at 37°C, 5% CO_2_ prior to inoculation from either apical or basolateral surface. Subsequent virus incubation, washing and sampling steps were performed as described above. HAE cultures from donor 59701 were used in duplo for 15 μg/ml αV ITG mAb experiments. Donors 48401, 43601, and 19702 were used as biological replicates for 30 μg/ml αV ITG mAb and heparin experiments. As described above for HAE cultures, identical αV ITG and HS blocking experiments were performed against HPeV1 in HT29 cell line (duplicate wells in 96-well microtiter plates). EV71 was used as a positive control for heparin experiments in RD cell line (Tan et al., 2013).

### Immunofluorescence imaging

HAE donor 43601 cultures were basolaterally inoculated with HPeV1 or HPeV3 (or mock) as described above, excluding the post-inoculation washing, incubated at 37°C, 5% CO_2_ for 12-h and fixed with 4% formalin in PBS for 30 min at room temperature (RT). HAE culture membranes were then excised from the inserts, submerged in 0.1% Triton X-100 for 15 min at RT for permeabilization and in 0.5% Tween20, and 10% bovine serum albumin (BSA) in PBS at 4°C overnight for blocking. HPeV1 and HPeV3 staining was performed using rabbit hyperimmune serum (kindly provided by Dr. Susi, University of Turku, Finland), which was raised against inactivated HPeV1 virions and shown to cross-react with HPeV3 (Joki-Korpela et al., 2000; Karelehto et al., 2017). Additional immunofluorescent labeling was performed using the following primary antibodies; mouse β-tubulin-Cy3 mAb (128K4872, Sigma Aldrich), mouse mucin 5B mAb (sc-393952; Santa Cruz, Dallas, TX), goat p63 pAb (AF1916; R&D Systems, Minneapolis, MN), αV ITG mAb clone L230 (ALX-803-304-C100; Enzo Life Sciences), and HS mAb clone F58-10E4 (370255-1; amsbio LLC, Cambridge, MA). Secondary antibodies used included donkey anti-rabbit IgG Alexa488 ab (A-21206; Life Technologies, Carlsbad, CA), donkey anti-mouse IgG Alexa546 ab (A10036; Life Technologies), donkey anti-goat IgG Alexa680 (A-21084; Life Technologies), donkey anti-rabbit IgG Alexa488 (A-21206; Life Technologies) and goat anti-mouse IgM Alexa555 (A-21426; Life Technologies). All antibodies were diluted in 0.5% Tween20 and 3% BSA in PBS. Cultures were incubated with primary antibodies at 4°C overnight and with secondary antibodies for 1-h at RT and washed extensively after each step with 0.5% Tween20 in PBS. Membranes were mounted on objective slides with ProLong Gold Antifade Mountant with DAPI (Life Technologies). Samples were examined by confocal immunofluorescence imaging using Leica TCS SP8 X microscope with HC Plan Apochromat 63x/1.40 oil objective and Leica LAS AF software (Leica Microsystems, Wetzlar, Germany). Confocal image stacks were deconvoluted by Huygens software package (SVI, Hilversum, the Netherlands) and subsequently compiled into 3D images and videos by Leica LAS AF.

### Microarrays pre-processing and normalization

HAE (donor 59701) cultures in five technical replicates were basolaterally inoculated with HPeV1 or HPeV3 (or mock) as described above and incubated at 37°C, 5% CO_2_. Cultures were lysed and RNA extracted by High Pure RNA Isolation Kit (Roche LifeScience, Penzberg, Germany) at the peak of replication 3 days post infection. RNA samples were concentrated to 33 ng/μl using Eppendorf Concentrator Plus (Eppendorf, Hamburg, Germany), and the concentration and quality was determined by Nanodrop (ThermoFisher Scientific) and BioAnalyzer (Agilent Technologies, Santa Clara, California). The extracted and concentrated total RNA samples were labeled and hybridized using the Human Clariom S HT microarray platform according to the manufacturer's guidelines (Affymetrix/ThermoFisher Scientific). The technical quality of arrays was assessed by arrayQualityMetrics R package (Kauffmann et al., 2009). All the samples, except one HPeV3 infected replicate, passed the quality control assessments. The total number of samples used in the analysis included 14 array replicates distributed as follows; 5 mock, 5 HPeV1-infected and 4 HPeV3-infected. The BrainArray (version 22) custom Chip Description File (CDF) was used to re-map probesets on the arrays based on the latest genome and transcriptome information (Dai et al., 2005). The FeatureFilter function, implemented in the geneFilter R package, was used to remove probesets which lacked their respective entrezID annotations (Bourgon et al., 2010). Probesets normalization was achieved by Robust Multi-array Average (RMA) function implemented in the Affy R package (Irizarry et al., 2003; Gautier et al., 2004). The final expression set comprised 18618 genes and 14 samples.

### Statistical analysis

For the HPeV replication kinetics, the relative increase in HPeV RNA copy numbers and HPeV TCID50 titers were calculated by subtracting the timepoint zero input values from all values. HPeV RNA copy number and TCID50 titer means of the technical and biological replicates between the apical and basolateral inoculation, or the mock and the mAb or heparin-treated samples, were compared per each virus and time point by two-way ANOVA with Tukey's multiple comparisons test using GraphPad Prism 7 (GraphPad Software Inc., La Jolla, CA).

Differentially expressed genes were identified using the limma R package (Ritchie et al., 2015). In brief, eBayes moderated t-statistics was applied to test 3 alternative hypothesis: (1) that gene expressions of HPeV1-infected HAE were different from mock, (2) that gene expressions of HPeV3-infected HAE were different from mock and (3) that gene expressions of HPeV1-infected HAE were different from HPeV3-infected HAE. For each test, we included in the empirical Bayes estimation a mean-variance trend, to account for probes that are less reliable at lower intensities. Nominal *P*-values of log2 fold changes were corrected for multiple testing using Benjamini-Hochberg False Discovery Rate (FDR). Differentially expressed genes were considered significant if their absolute fold change was greater than 1.2 and the associated FDR adjusted p value was below 5e-2.

Gene Ontology over-representation analyses of biological processes described by the selected DEGs were performed using the topGO R package (Alexa et al., 2006). Significance for each individual GO-term was computed by means of the weight01 algorithm and Fisher's exact test.

## Results

### Human parechovirus infection of the airway epithelium is polarized

**Figure 1:**
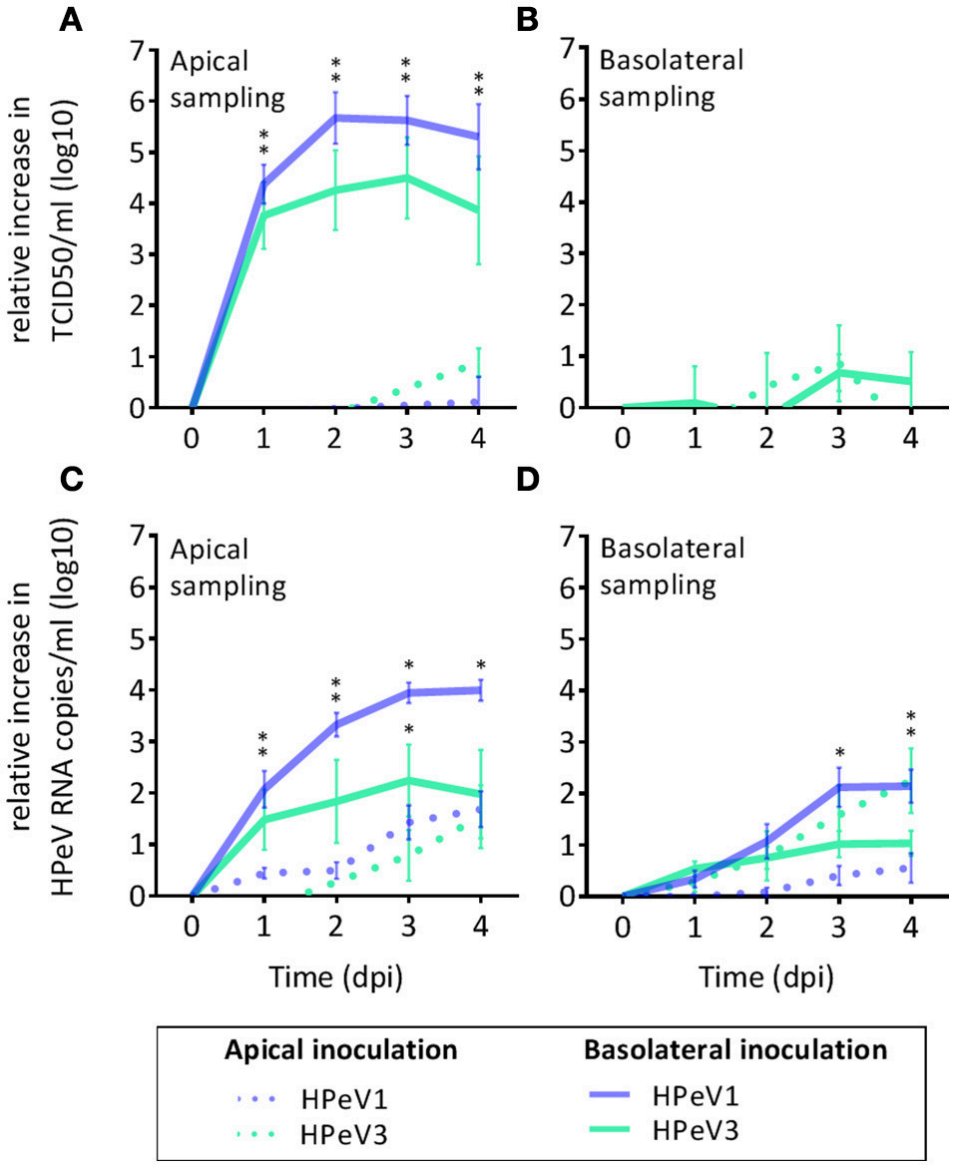
HPeV1 and HPeV3 replication kinetics in nasal HAE cell cultures. Relative increase in HPeV 50% tissue culture infectious dose (TCID50) titers in the apical **(A)** and basolateral **(B)** HAE samples, and relative increase in the HPeV RNA copies determined by RT-qPCR analysis in the apical **(C)** and basolateral **(D)** samples following either apical (dotted line) or basolateral inoculation (solid line) of the HAE cultures. Data represents the mean and standard error of mean for four technical and four biological replicates. *p-values <4e-4. Dpi; days post infection.

To investigate HPeV replication in the human airway epithelium, we inoculated well-differentiated nasal HAE cultures, established from four individual donors, with HPeV1 and HPeV3 either from the apical or the basolateral surface (Figure 1). HPeV1 and HPeV3 could both replicate in HAE cultures. Apical inoculation resulted in low virus titers whereas basolateral inoculation led to significantly higher HPeV1 and HPeV3 titers at the apical compartment 1–3 days post infection (dpi; *p*-values <4e-4) (Figure 1A). Infectious virus titers detected from the basolateral compartment were low with no significant difference between the apical and basolateral inoculation (Figure 1B). TCID50 analyses showed that the viral replication peaked at day 2 or 3 (Figure 1A). RT-qPCR analyses of the apical samples were in line with TCID50 titrations (Figure 1C). Significant increases in the viral RNA at the basolateral compartment was detected following apical inoculation (Figure 1D). However, this was not reflected in the amount of infectious virus (Figure 1B). TCID50 data could not be used to directly compare HPeV1 and HPeV3 replication efficiencies due to the different cell lines used in the assays. However, relative increase in the HPeV1 RNA copy numbers were significantly higher 2 to 4 dpi (basolateral inoculation and apical sampling, *p*-values <1e-4) and 3 to 4 dpi (basolateral inoculation and basolateral sampling, *p*-values <4e-2), whereas relative increase in the HPeV3 RNA copy numbers were significantly higher 3 to 4 dpi (apical inoculation and basolateral sampling, *p*-values <2e-2). Efficient apical infection in the HAE cultures could be established with the respiratory picornavirus enterovirus 68 (EV68; data not shown).

### HPeVs preferentially infect p63 positive basal cells in the airway epithelium

**Figure 2:**
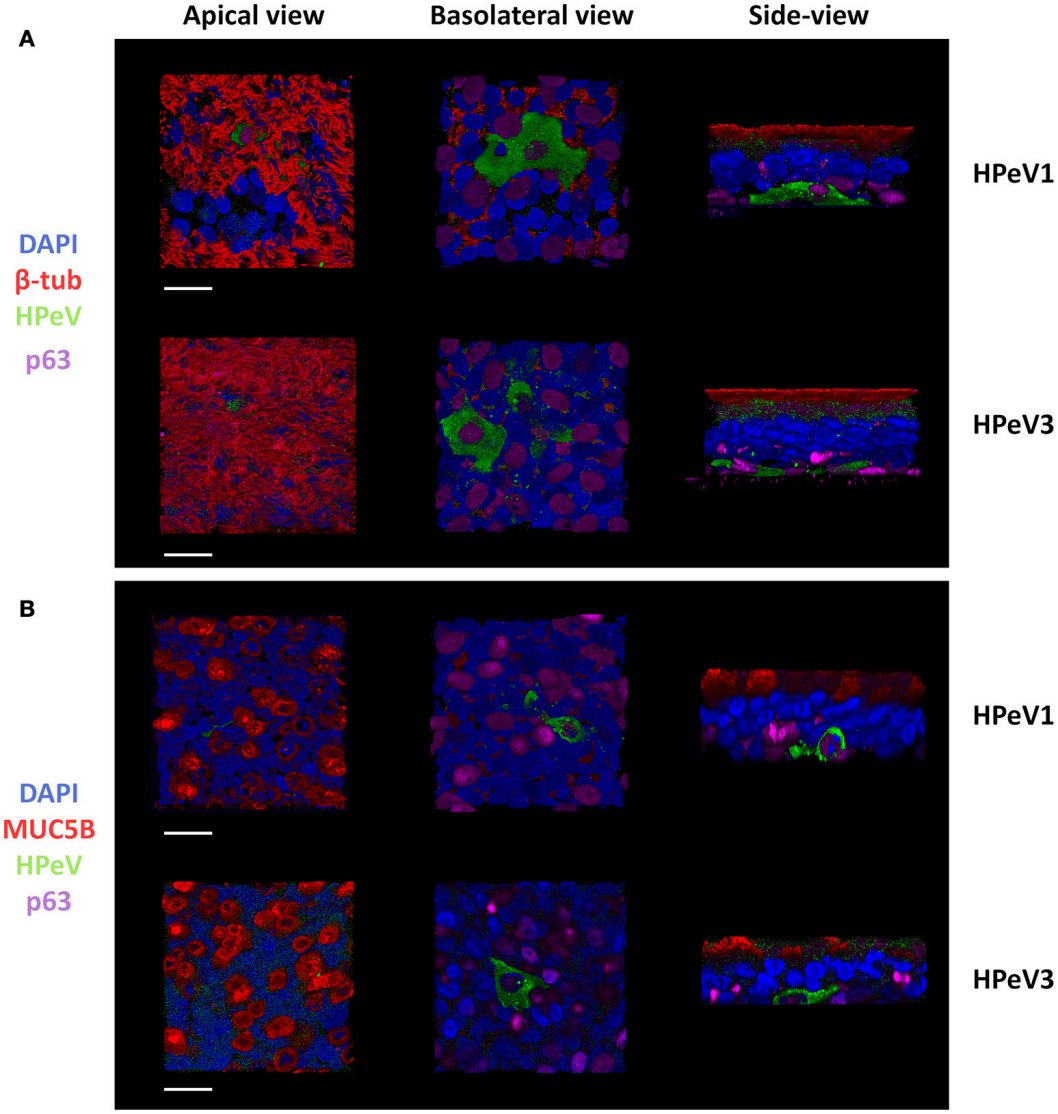
Human parechoviruses target p63 positive basal cells in the airway epithelium. Confocal image stacks of HPeV1- and HPeV3-infected HAE cultures triple-stained by **(A)** ciliated cell marker β-tubulin (red), basal cell marker p63 (purple) and HPeV antibody (green) or **(B)** secretory cell marker mucin 5B (red), basal cell marker p63 (purple) and HPeV antibody (green). Nuclei stained by DAPI (blue). Scale bars, 20 μm.

To determine the HPeV target cell type, we performed immunofluorescent labeling and confocal microscopy of the HAE cultures. Triple-staining of the HPeV1- and HPeV3-infected HAE cultures at 12-h post inoculation by ciliated cell marker β-tubulin, basal cell marker p63, and HPeV antibody (Figure 2A) or secretory cell marker MUC5B, basal cell marker p63, and HPeV antibody (Figure 2B) revealed that while ciliated and secretory cells were localized on the apical surface of the HAE culture, HPeV-infected cells resided near the basolateral surface and were positive for the p63 basal cell marker (Figure 2). 3D renderings of the confocal image stacks are provided in Supplementary videos 1–6. In addition, we observed that αV integrins were clearly localized to the basolateral surface of the HAE whereas heparan sulfate was diffusely stained throughout the cultures (Supplementary Figure 1).

### Blocking of αV integrins or heparan sulfate does not inhibit HPeV replication in HAE model

**Figure 3:**
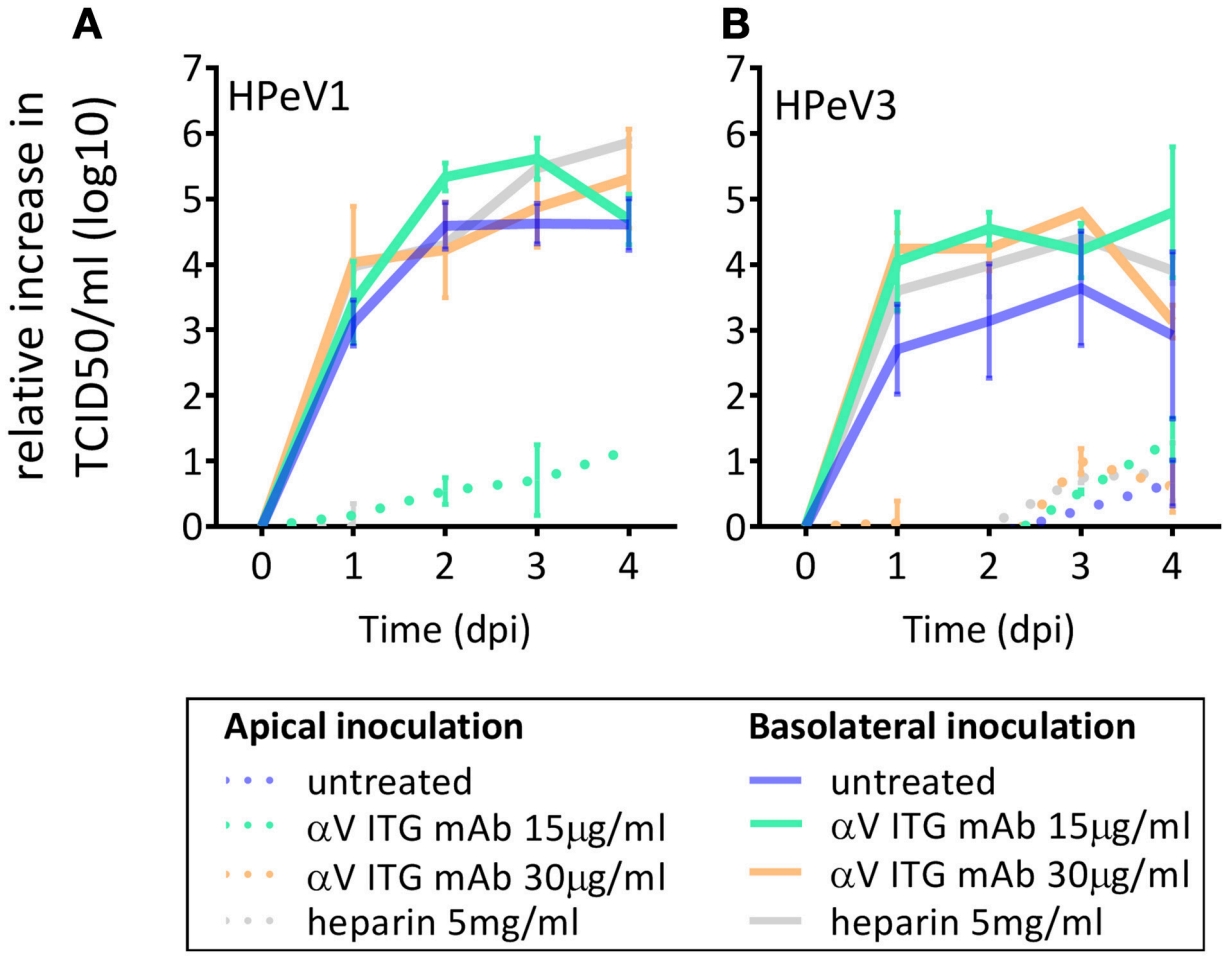
Effect of αV ITG and HS blocking on HPeV replication. Relative increase in HPeV1 **(A)** and HPeV3 **(B)** 50% tissue culture infectious dose (TCID50) titers in apical samples following function blocking ITGαV mAb pretreatment (15 μg/ml or 30 μg/ml) of the HAE cultures or heparin pretreatment (5 mg/ml) of the virus inoculum. Mean and standard error of mean for three biological replicates is shown. Basolateral sampling data not shown.

As αV ITGs and HS have been described to function as HPeV1 receptors *in vitro*, we investigated the effect of blocking αV ITGs on either apical or basolateral surface of the HAE model prior to HPeV1 and HPeV3 inoculation. Neither of the tested concentrations of the function-blocking αV ITG monoclonal antibody (mAb) significantly inhibited HPeV1 (Figure 3A) or HPeV3 (Figure 3B) replication. To block HS binding sites on the virus capsid, we incubated the viruses with heparin prior to either apical or basolateral HAE inoculation but detected no significant inhibition of either HPeV1 (Figure 3A) or HPeV3 replication (Figure 3B).

HPeV1 replication was inhibited in HT29 cell line following αV ITG blocking, albeit this did not reach statistical significance (Supplementary Figure 2). However, we could not confirm the role of HS in HPeV1 infection in HT29 (Supplementary Figure 2), while functionality of the same concentration of heparin in blocking was shown by complete inhibition of enterovirus 71 (EV71) replication in the RD cell line (data not shown). Due to the poor replication HPeV3 in standard cell culture, we could not perform similar receptor blocking for HPeV3.

### HPeV1 and HPeV3 induce distinct transcriptional profiles in the airway epithelium

**Figure 4:**
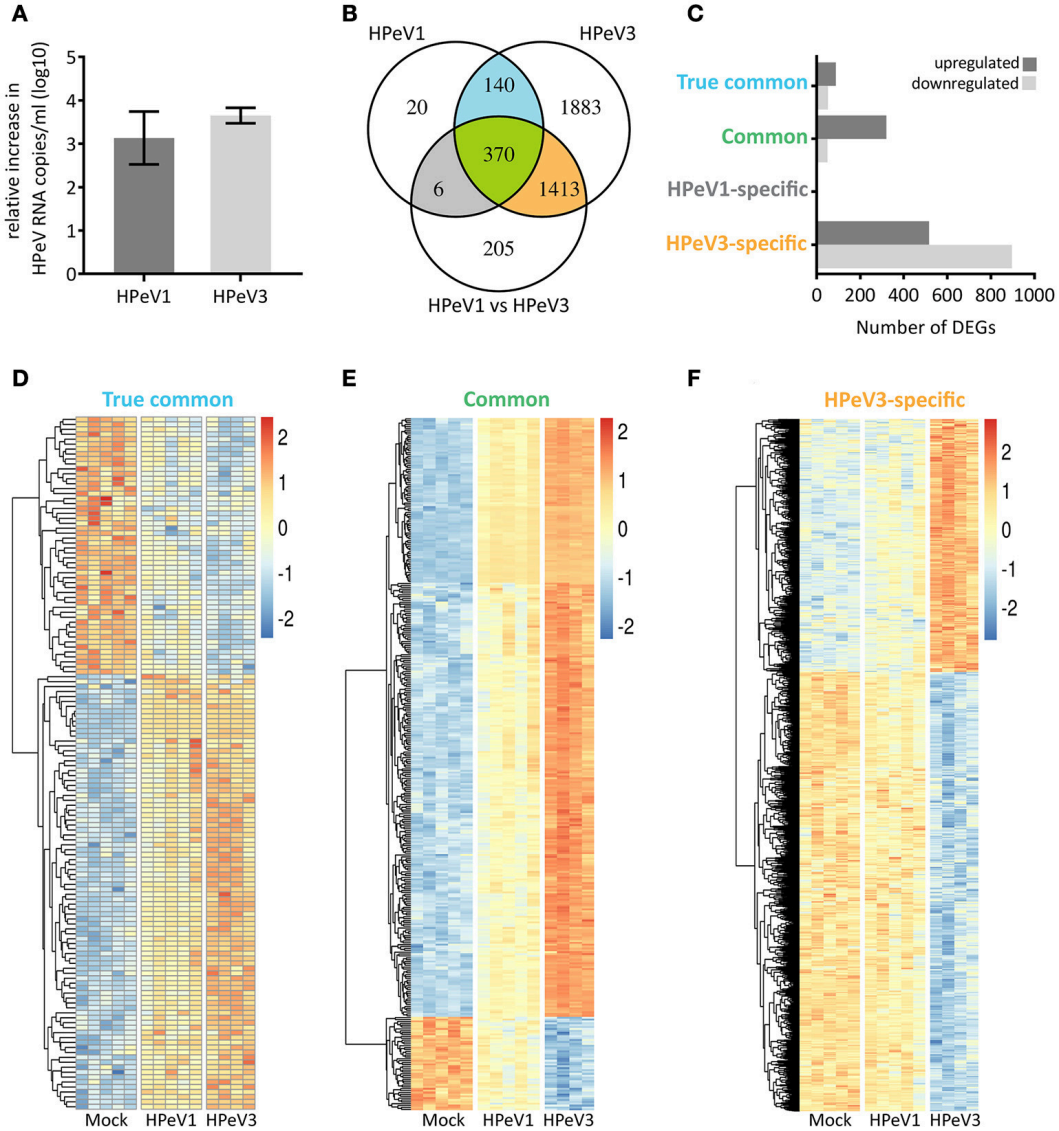
Overview of the significantly differentially expressed genes (DEGs) induced by HPeV infection at day 3 post infection (*p* < 0.05, gene expression fold change >1.2 log2). **(A)** RT-qPCR analysis of HPeV replication in the HAE apical compartment at the time of microarray sample preparation. **(B)** Venn diagram showing the total number, overlap and differences in the DEGs induced by HPeV1 and HPeV3. **(C)** Number of up- and downregulated genes among the four subsets of DEGs; true common, common, HPeV1-specific and HPeV3-specific. **(D)** Heatmap of the true common DEGs induced similarly by both HPeV1 and HPeV3. **(E)** Heatmap of the common DEGs induced differently by HPeV1 and HPeV3. **(F)** Heatmap of the DEGs induced exclusively upon HPeV3 infection. Gene expression fold change scale in the heatmaps is normalized by row values.

Next, we performed transcriptome analyses to compare the airway epithelium host response against HPeV1 and HPeV3 infection. Virus replication in the HAE was confirmed by RT-qPCR (Figure 4A). The total numbers and overlap in significantly differentially expressed genes (DEGs; adj. *p*-value <5e-2, gene expression fold change >1.2) are depicted in the Venn diagram (Figure 4B). As compared to the mock, HPeV1 induced differential expression of 536 genes whereas HPeV3 induced 3806 genes. Of these, 510 genes were shared among the two viruses (Figure 4B). Of the shared genes, the expression profiles of 140 genes did not significantly differ between HPeV1 and HPeV3. Thus, these genes represent a true common HPeV DEG signature. 370 of the shared genes were found to be differentially expressed upon both HPeV1 and HPeV3 infection, but to a significantly different level. These genes represent the common HPeV response. Remarkably, only 6 DEGs were specific to HPeV1 in contrast to 1413 DEGs found to be induced exclusively by HPeV3. Of the true common DEGs, 88 were upregulated and 52 downregulated when compared to the mock (Figure 4C). Common DEGs included 320 upregulated and 50 downregulated genes, while 516 of HPeV3-specific DEGs were upregulated and 897 downregulated. Heatmaps of the true common, common, and HPeV3-specific DEGs are presented in Figures 4D–F. Direction of the gene expression among the true common and common DEGs was identical regardless of the HPeV genotype (Figures 4D,E). Overall HPeV3 infection resulted in greater fold change of the common transcripts (Figure 4E). HPeV3-specific DEGs were either up- or downregulated upon HPeV3 infection whereas expression of these genes remained at baseline in HPeV1 infection (Figure 4F). Complete lists of all the DEGs induced by HPeV1 and HPeV3, and a list of the DEGs different between HPeV1 and HPeV3, are provided in Supplementary Tables 1–3.

### HPeV3 infection results in robust immune and inflammatory responses

**Figure 5:**
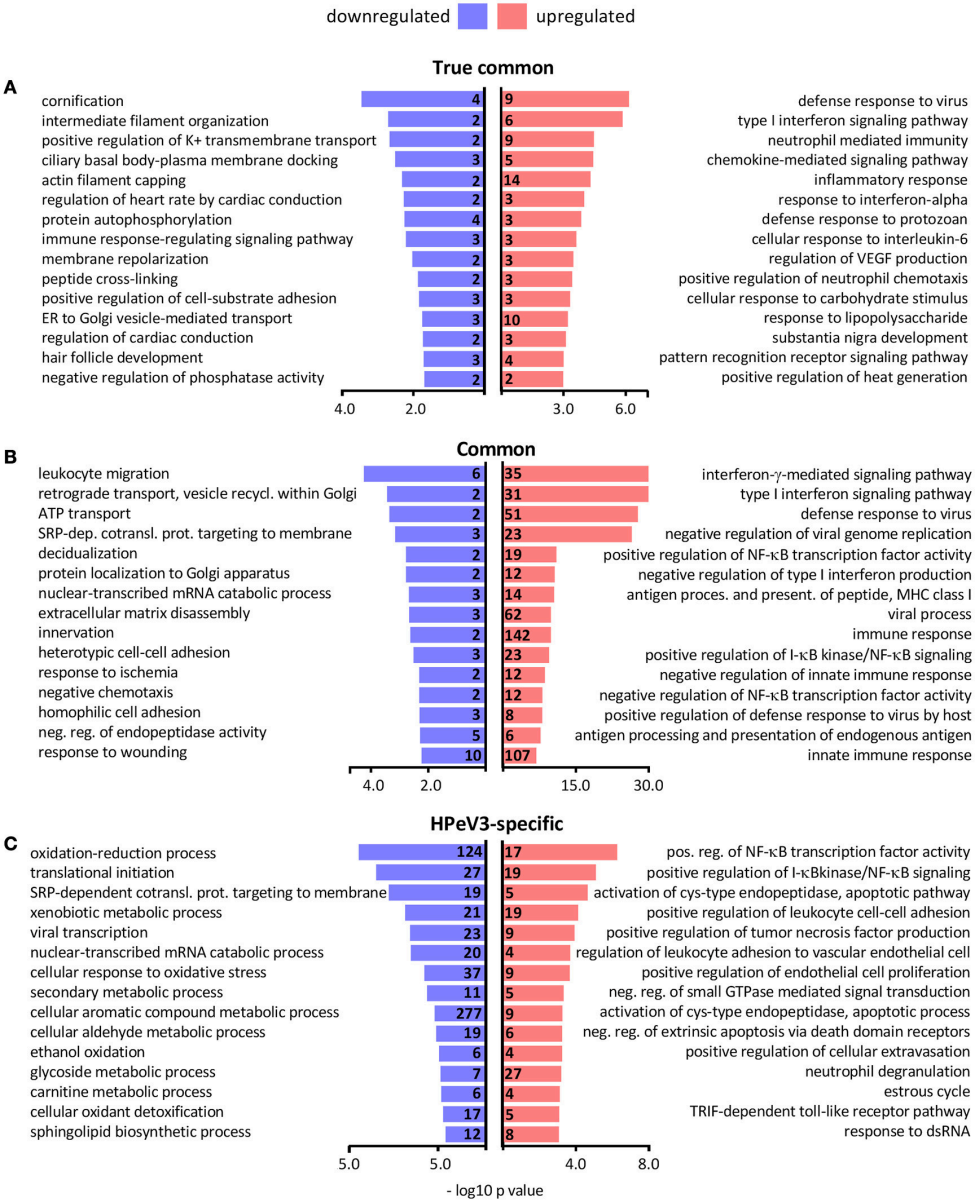
Top 15 most significantly over-represented biological processes among the gene ontologies of the **(A)** true common, **(B)** common and **(C)** HPeV3-specific up- and downregulated DEG subsets. Number of DEGs associated with each biological process shown along y-axis and -log10 *p*-values on the x-axis.

Gene ontology term over-representation analysis was performed to investigate which biological processes are associated with the observed DEGs. As expected the biological processes enriched in the true common set of upregulated genes were almost exclusively related to immune response and inflammation, whereas the few downregulated DEGs were associated with processes such as epithelial differentiation (cornification) and cytoskeleton remodeling (intermediate filament organization, actin filament capping) (Figure 5A). Hundreds of common DEGs significantly more upregulated by HPeV3 than by HPeV1 were enriched for genes involved in immune responses and inflammation (type I and II interferon responses, NF-κB signaling) (Figure 5B). Many of the common DEGs that were more downregulated by HPeV3 than by HPeV1, were related to cell adhesion (leukocyte migration, extracellular matrix disassembly, heterotypic cell-cell adhesion, response to wounding).

Only six genes were found to be differentially expressed exclusively in HPeV1 infection. Three of these genes were downregulated (alpha kinase 3 (ALPK3), MSS51 mitochondrial translational activator (MSS51), biorientation of chromosomes in cell division 1 like 1 (BOD1L1) (Fold changes −1.4, −1.6, −1.3 and adj. *p*-values 1e-2, 8e-3, 1e-2, respectively)), and three were upregulated (ATPase phospholipid transporting 8B2 (ATP8B2), tensin 3 (TNS3), matrix metallopeptidase 7 (MMP7) (Fold changes 1.5, 1.4, 1.8, and adj. p values 3e-2, 4e-2, 2e-3, respectively)) (Supplementary Table 1). HPeV3-specific upregulated genes were most notably enriched in the NF-κB signaling pathway activation (Figure 5C). HPeV3 infection increased the expression of key molecules involved in this pathway such as proto-oncogene RelB, interleukin 1 receptor associated kinase 4 (IRAK4) and TNF receptor associated factor 2 (TRAF2) (Fold changes 2.5, 1.6, 1.3, and adj. *p*-values 6e-6, 4e-5, 1e-3, respectively) (Supplementary Table 2). Other HPeV3-specific enriched processes included for example tumor necrosis factor signaling and leukocyte adhesion (Figure 5C). Each of the viruses induced both up- and downregulation of a number of apoptosis-associated genes (Supplementary Tables 1, 2). However, HPeV3-specific upregulated DEGs were significantly enriched for apoptosis-related processes such as activation of cysteine-type endopeptidase and actin filament organization (Figure 5C). For example, of the essential apoptosis effectors, caspases 8 and 10 were significantly more upregulated by HPeV3 than by HPeV1 (Fold changes HPeV3 vs. HPeV1 1.5, 2.1, and adj. *p*-values 1e-2, 8e-5, respectively) (Supplementary Table 3).

The downregulated HPeV3-specific DEGs were annotated with various processes such as ones involved in protein translation (translational initiation, SRP-dependent cotranslational protein targeting to membrane), RNA degradation (nuclear-transcribed mRNA catabolic process) and cell metabolism (oxidation-reduction process, mitochondrial ATP synthesis coupled proton transport) (Figure 5C).

## Discussion

In this study we showed that the airway epithelium can support HPeV replication, that infection occurs preferentially from the basolateral surface, and that the progeny virus is released from the apical surface of the epithelium. Moreover, we identified p63+ basal cells as the HPeV target cell type in the HAE model and found that the magnitude of innate immune responses was greater following HPeV3 than HPeV1 infection.

In contrast to what has been established previously for several respiratory viruses such as influenza virus, rhinovirus (RV), coronavirus, or respiratory syncytial virus (RSV) (Matrosovich et al., 2004; Johnson et al., 2015; Griggs et al., 2017), HPeVs infected airway epithelium more efficiently from the basolateral rather than the apical surface. Similarly, basolateral entry in the HAE cultures has been reported for vaccinia virus, reovirus, some adeno- and arenaviruses and measles virus (Sinn et al., 2002; Vermeer et al., 2007; Dylla et al., 2008; Excoffon et al., 2008; Lütschg et al., 2011). In line with our HPeV data, vaccinia, reo-, and measles viruses also exhibited apical shedding of the progeny virions (Sinn et al., 2002; Vermeer et al., 2007; Excoffon et al., 2008). We found that both HPeV1 and HPeV3 targeted the p63+ basal cells for replication. Basal cells are the progenitor cell type of the airways and reside on the basolateral surface underneath a layer of differentiated ciliated and secretory cells (Rock et al., 2009). Tropism toward the p63+ cells therefore explains the basolateral polarity of HPeV infection. Similar tropism has been reported previously for RV (A species) and RSV, but only upon exposing the basal cells to apical inoculation by mechanical or chemical damage (Jakiela et al., 2008; Persson et al., 2014). Since basal cells maintain the epithelial integrity and regulate the proliferation, extensive infection of these cells could result in epithelial damage and thus lead to respiratory symptoms *in vivo*. However, in contrast to the infection in immortalized cell lines, we did not observe major cytopathic effects in HPeV1- or HPeV3-infected HAE cultures. Here we examined the HPeV cell tropism at 12-h post infection. Further experiments investigating later timepoints are necessary to determine whether cells other than those positive for the p63 marker can be infected.

Previously reported transcriptome analysis of the airway basal cells has showed that while αV ITG is not significantly more expressed in basal vs. the differentiated epithelia cells, β1 and β6 ITGs do belong to the specific basal cell transcriptome signature (Hackett et al., 2011). These β ITG subunits dimerize with αV ITG and have been described to be the high affinity receptors of HPeV1 in cell culture (Seitsonen et al., 2010; Merilahti et al., 2016b). HPeV1, but not HPeV3, contains the integrin-binding RGD-motif (Stanway et al., 1994; Ito et al., 2004), yet both viruses were found to infect the same cell type. Reports of basolateral localization of the proposed HPeV1 receptors, ITGs and HS (Erlinger, 1995; Lütschg et al., 2011; Johnson et al., 2015), prompted us to study the polarity of HPeV infection. However, we could only confirm basolateral localization of the αV ITGs but not of HS in HAE cultures. Blocking these receptors on either apical or basolateral surface of the HAE cultures had no inhibitory effect on either HPeV1 or HPeV3 replication. This is surprising since deletion of the RGD motif has been shown to abolish HPeV1 infectivity in cell lines (Boonyakiat et al., 2001). While we cannot exclude the possibility that the efficiency of the function-blocking αV ITG mAb pretreatment of the HAE or the heparin-pretreatment of the virus was insufficient, we did observe inhibition of HPeV1 in HT29 cell line upon identical αV ITG mAb pretreatment and successfully used EV71 as a positive control in our HS blocking experiments. Moreover, similar αV ITG mAb and heparin concentrations have been reported to inhibit HPeV1 replication in monolayer cell cultures (Triantafilou et al., 2000; Merilahti et al., 2016a,b). We believe differences in the cell lines and read-out methods may explain why we could not validate the previously reported heparin-blocking of HPeV1 in standard cell culture (Merilahti et al., 2016a). In summary, we speculate that the HPeV receptor-usage is cell line-dependent and may differ between monolayer cell cultures and polarized primary cell culture models such as HAE. Further research is needed to confirm this hypothesis.

Transcriptome analyses revealed that HPeV3-infection resulted in the expression of a considerable number of host genes whereas the HPeV1 response was relatively modest. Major difference between the responses was the over-representation of transcripts associated with immune and inflammatory responses following HPeV3 infection. HPeV1 and HPeV3 induced genes involved in type I and II interferon signaling and NF-κB pathways but the magnitude of these responses was higher in the HPeV3-infected HAE cultures. As reported by us and others, in contrast to the humoral immunity against HPeV1, the maternal neutralizing antibody protection against HPeV3 is low in women of childbearing age and lacking in HPeV3-infected newborns (Karelehto et al., PIDJ in press)(Aizawa et al., 2015). Therefore, innate immune response at the virus entry site could contribute to the severe clinical presentation. Two mechanisms may explain why HPeV3 more often than HPeV1 causes severe disease (Romero and Selvarangan, 2011). (i) Excessive release of proinflammatory cytokines following HPeV3 infection could result in immunopathology involving increased vascular permeability and compromised blood-brain-barrier integrity (Rochfort and Cummins, 2015; Danielski et al., 2018). This is perhaps supported by the association of HPeV3 and neonatal sepsis, a disease with various unspecific symptoms characterized by a systemic inflammatory response syndrome (Shane et al., 2017). (ii) Conversely, neonates are known to have immature innate immune system, including downregulation of genes involved in NF-κB signaling and decreased cytokine production (Raymond et al., 2017), and thus may fail to elicit the necessary host response to control HPeV3 infection. A detailed characterization of the host transcriptome by analyzing earlier timepoints and comparing different tissues from infants and adults, as well as validating the results at the protein level, will further elucidate the differences between HPeV1 and HPeV3 infection.

For measles virus a model of infection has been proposed in which the virus is taken up by alveolar immune cells, transported across the airway epithelium and into the lymphoid tissue, the site of primary replication, followed by systemic spread and basolateral infection combined with apical egress in the airway epithelium (Mühlebach et al., 2011). Recently, adenovirus 5 (AdV5) was shown to promote re-localization of its coxsackievirus and adenovirus receptor (CAREx8), via inducing IL8 secretion of the immune cells, thus enabling apical entry (Kotha et al., 2015). Similarly, we hypothesize that the airway-resident dendritic cells and macrophages play a role in facilitating HPeV entry into the polarized epithelium. Another explanation as to how HPeVs might infect the airway epithelium *in vivo* involves M-cells, a specialized type of epithelial cells overlaying the Peyer's patches in the intestinal epithelium (Mabbott et al., 2013). Poliovirus, as well as the reovirus, has been shown to break the epithelial barrier by transcytosis through M-cells (Morin et al., 1994; Ouzilou et al., 2002; Gonzalez-Hernandez et al., 2014). Similar type of antigen-sampling cell has also been described in the airway epithelium (Morin et al., 1994; Mabbott et al., 2013).

While HAE cell culture system is an elegant model, it contains only epithelial cells. Underlying mesenchymal cells such as fibroblasts and immune cells can modulate the epithelial function and likely play a role in HPeV pathogenesis (Nowarski et al., 2017). Co-culture systems incorporating various cell types are more suitable to answer questions such as how HPeVs access airway epithelium basal cells *in vivo*. In addition, as HPeVs can invade the CNS (Romero and Selvarangan, 2011), and as many of the related enteroviruses gain entry in the human gut (Semler and Wimmer, 2002), organoids represent attractive models to further study HPeV infection of the CNS and intestinal epithelium (Iakobachvili and Peters, 2017).

In conclusion, we showed that the HAE is permissive to HPeV infection, that HPeV1 and HPeV3 infect it in a polarized manner by targeting the basal progenitor cells, and that HPeV3, the genotype causing outbreaks of severe illness in neonates, induces an amplified immune and inflammatory host response when compared to HPeV1. This work represents the first step toward understanding the entry and genotype-dependent pathogenesis of human parechoviruses.

## Data availability

The data discussed in this publication has been deposited in NCBI's Gene Expression Omnibus (Edgar et al., 2002) and is accessible through GEO Series accession number GSE117183 (https://www.ncbi.nlm.nih.gov/geo/query/acc.cgi?acc=GSE117183). Authors: Cosimo Cristella, Eveliina Karelehto, Xiao Yu, Katja C. Wolthers, Menno D. de Jong. Year: 2018. Title: Gene expression data of human airway epithelium following human parechovirus infection.

## Author contributions

EK, AS, and KW designed the experiments. EK, XY, AS, RH, KdH, HvE, and SK performed all experimental work. CC performed the transcriptome analyses. EK wrote the first draft of the manuscript. All authors contributed to manuscript revision, read, and approved the final version.

### Conflict of interest statement

The authors declare that the research was conducted in the absence of any commercial or financial relationships that could be construed as a potential conflict of interest. The reviewer KG and handling Editor declared their shared affiliation.
