# Supplementary figures and images for: Polarized Entry of Human Parechoviruses in the Airway Epithelium
Source: Front Cell Infect Microbiol. 2018 Aug 22;8:294. doi: 10.3389/fcimb.2018.00294 (PMC6119779; doi:10.3389/fcimb.2018.00294)

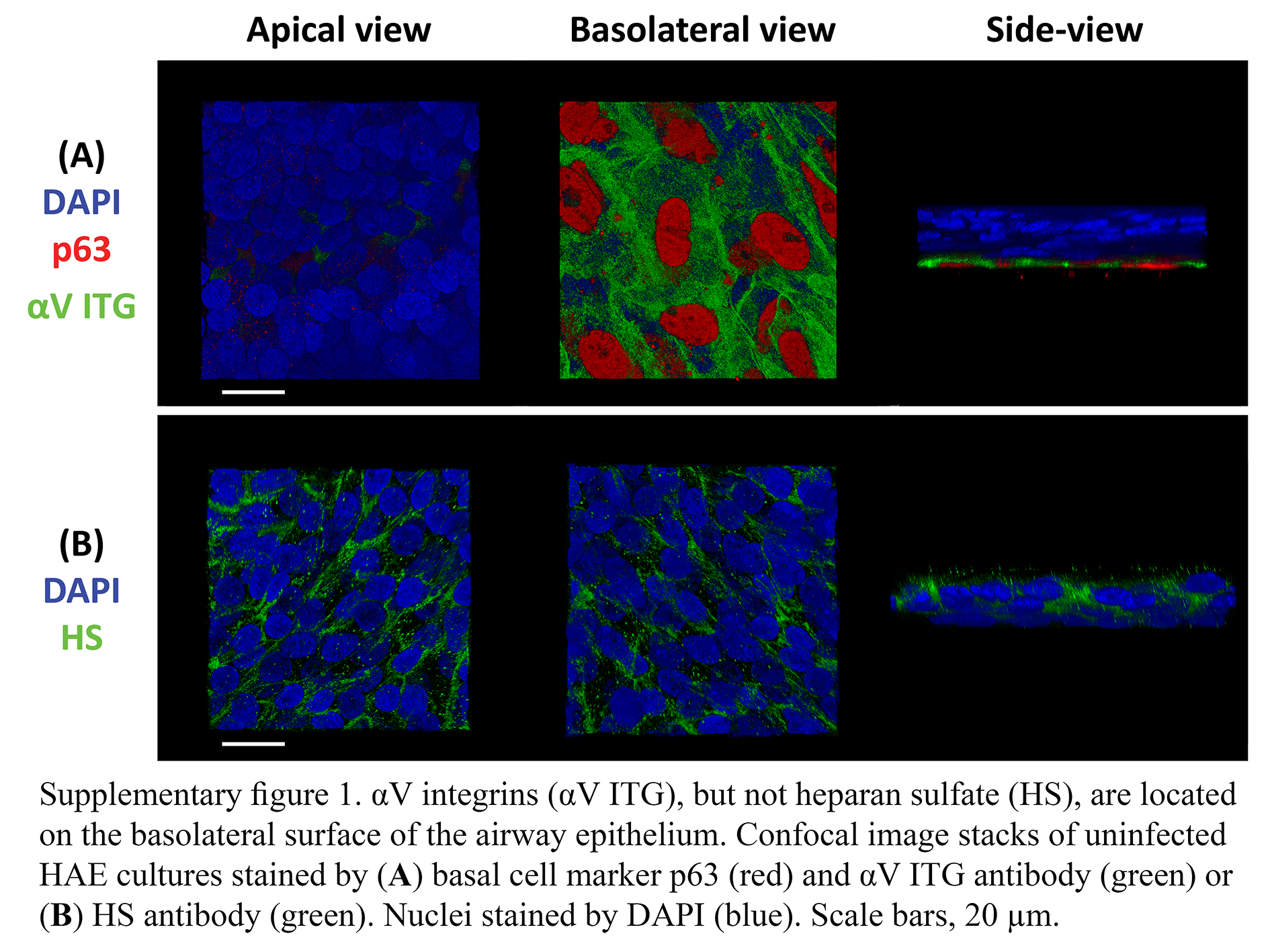

Supplement: Supplementary file 1 [file Image_1.TIF]

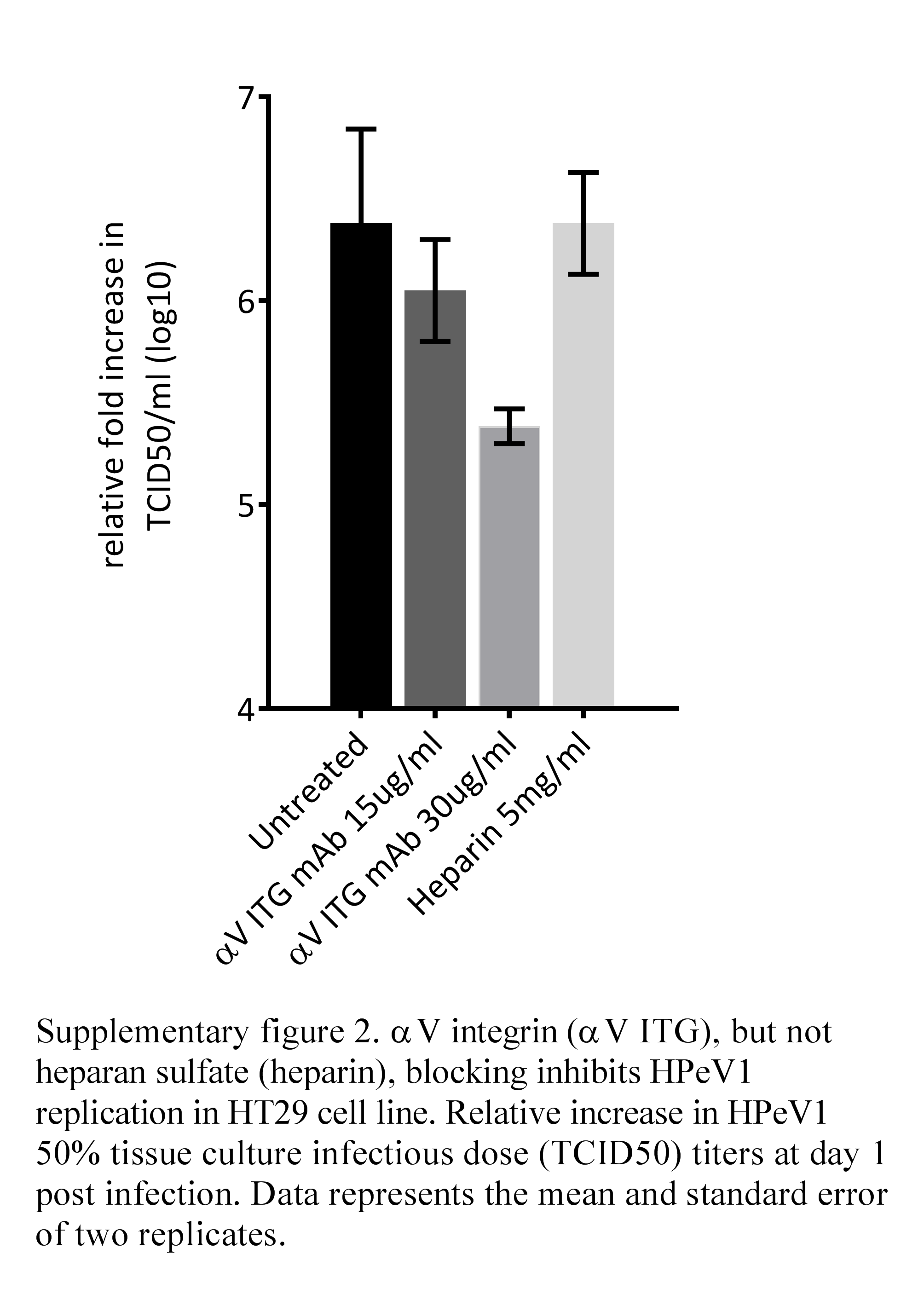

Supplement: Supplementary file 2 [file Image_2.TIF]
